# Supplementary material for: RMD and Its Suppressor MAPK6 Control Root Circumnutation and Obstacle Avoidance via BR Signaling
Source: Int J Mol Sci. 2024 Sep 30;25(19):10543. doi: 10.3390/ijms251910543 (PMC11477179; doi:10.3390/ijms251910543)
Supplement: Supplementary file 1 [file ijms-25-10543-s001.zip › ijms-3223931-Supplementary figure and table-Revised2024.9.27.pdf]

Supplemental information

RMD and its suppressor MAPK6 control root circumnutation and obstacle avoidance via BR signaling

Le Dong*, Dongdong Liu, Shiyu Sun, Jianxin Shi, Staffan Persson, Guoqiang Huang, Dabing Zhang

Contents

Figure S1. In-gel root growth

Figure S2. Schematic representation of primary root growth in different genotypes during circumnutation

Figure S3. MAPK6 protein levels in 7 d WT, *rmd*, *rmd sor8*, and *sor8* roots

Figure S4. Knocking out of *RMD* in the BZR1-GFP marker line

Figure S5. Expression of BR feedback-regulated genes in 7 d WT and *rmd* roots

Figure S6. Melting curve analysis of qRT-PCR reactions for check of specificity of the primers used for *RMD* expression analysis under BL treatment in Figure 5C.

Figure S7. CRISPR/Cas9–induced mutation of *BRI1*, *D2*, *BIM2, MKK4* in the *rmd* background

Figure S8. The relative expression of *GSK2* in representative *GSK2* overexpression lines in *rmd* background

Figure S9. Root phenotypes of WT, *rmd*, and the transgenic T_0 line of *mkk4 rmd* lines

Figure S10. BIM2 positively regulates BR signaling

Figure S11. Gravity response of WT, *rmd*, *rmd sor8*, and *sor8* lines

Table S1. Primers used in this study

Supporting movies legends

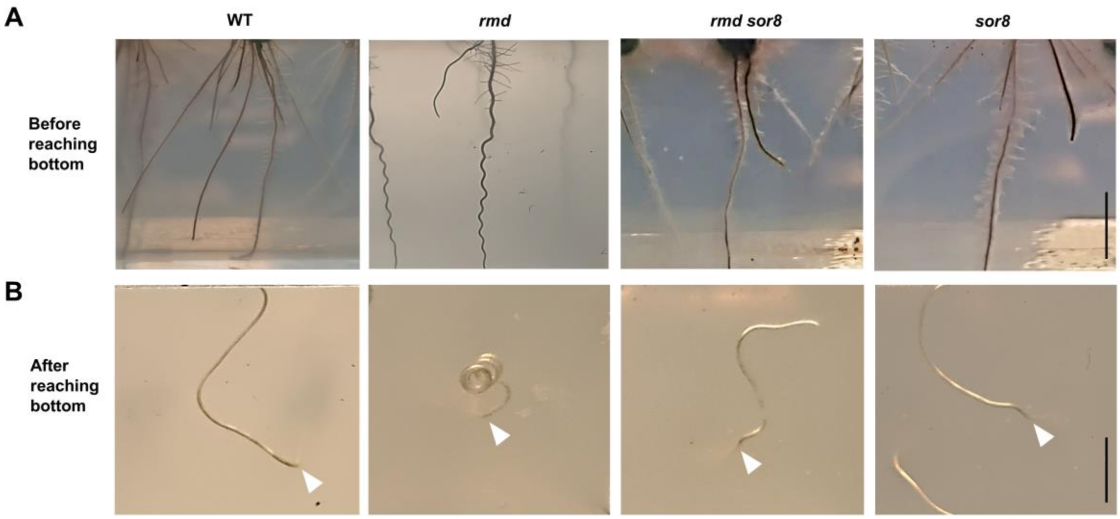

Figure S1. In-gel root growth

(A) Primary root of WT, *rmd*, *rmd sor8*, and *sor8* growing vertically in MS agar (pH 5.8). Scale bars, 1 cm. **(B)** Primary root growth after reaching the container bottom; white triangles indicate site of first contact. Photos were taken from the bottom of the container. Scale bars, 0.5 cm.

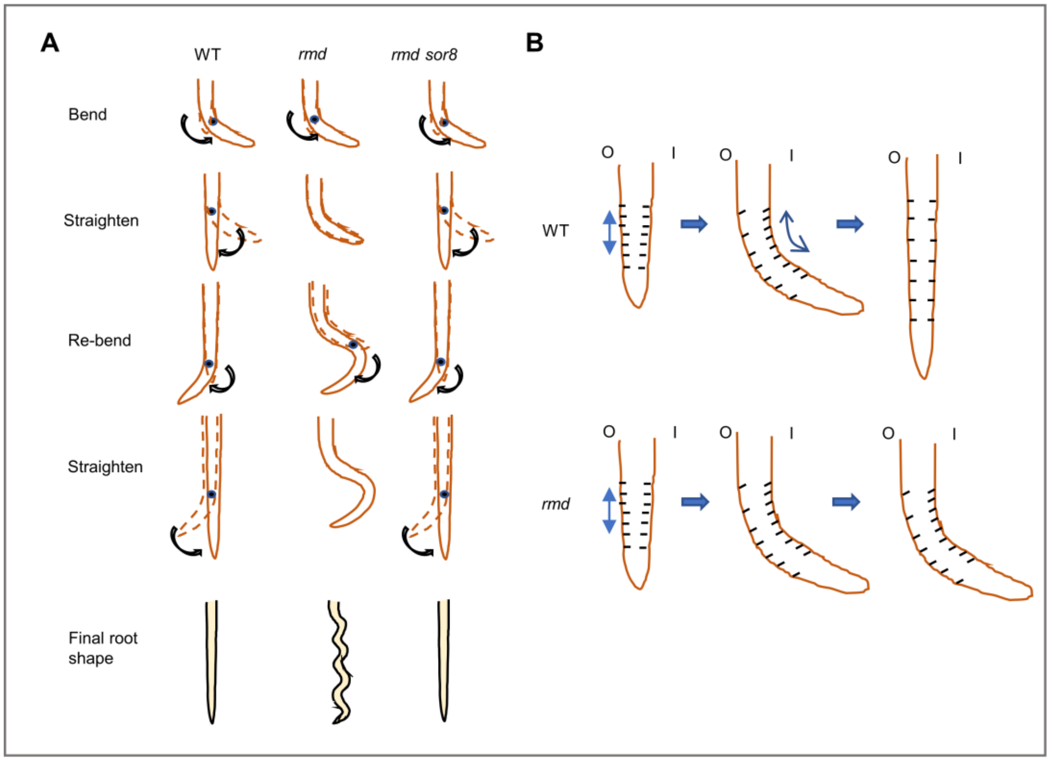

Figure S2. Schematic representation of primary root growth in different genotypes during circumnutation

(A) In WT and *rmd sor8* lines, primary roots bend (circumnutate) to one side, then straighten before next bend occurs; the final root is straight. In *rmd* lines, the primary root bends from side to side at the root tip without an intermediate straightening step. **(B)** In WT roots, outer cells (O) elongate to form a bend, then the inner cells (I) elongate immediately afterwards, straightening the root. In *rmd* roots, the inner cells cannot elongate to straighten the root after a bend.

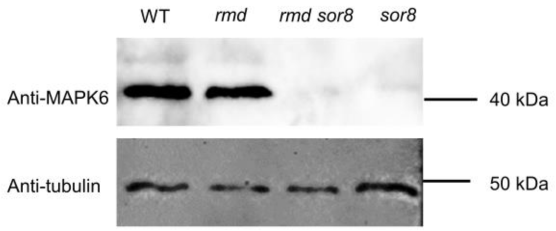

Figure S3. MAPK6 protein levels in 7 d WT, *rmd*, *rmd sor8*, and *sor8* roots

Anti-tubulin is shown as loading control.

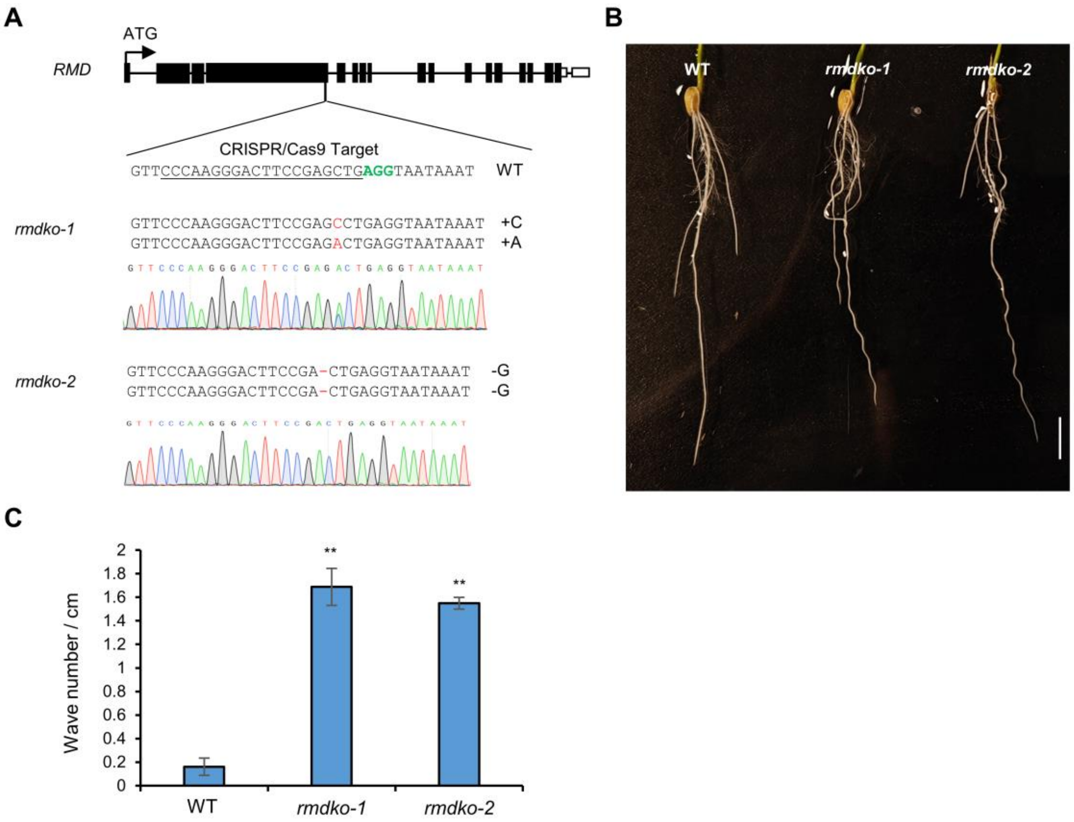

Figure S4. Knocking out of *RMD* in the BZR1-GFP marker line

(A) CRISPR/Cas9–induced mutation of *RMD*. Black boxes show exons; black lines introns; and open boxes untranslated regions. 20 bp gRNAs are underlined and PAMs highlighted in green. Mutations in resultant lines are highlighted in red. **(B)** Phenotype of *rmdko* lines. Scale bar, 1 cm. **(C)** Statistic data of wave number per unit root length in WT and BZR1-GFP *rmdko* T_1 lines. Data are means ± s.d. (n = 3 biological replicates). Asterisks indicate significant differences relative to WT (Student's *t*-test; **$p < 0.01$).

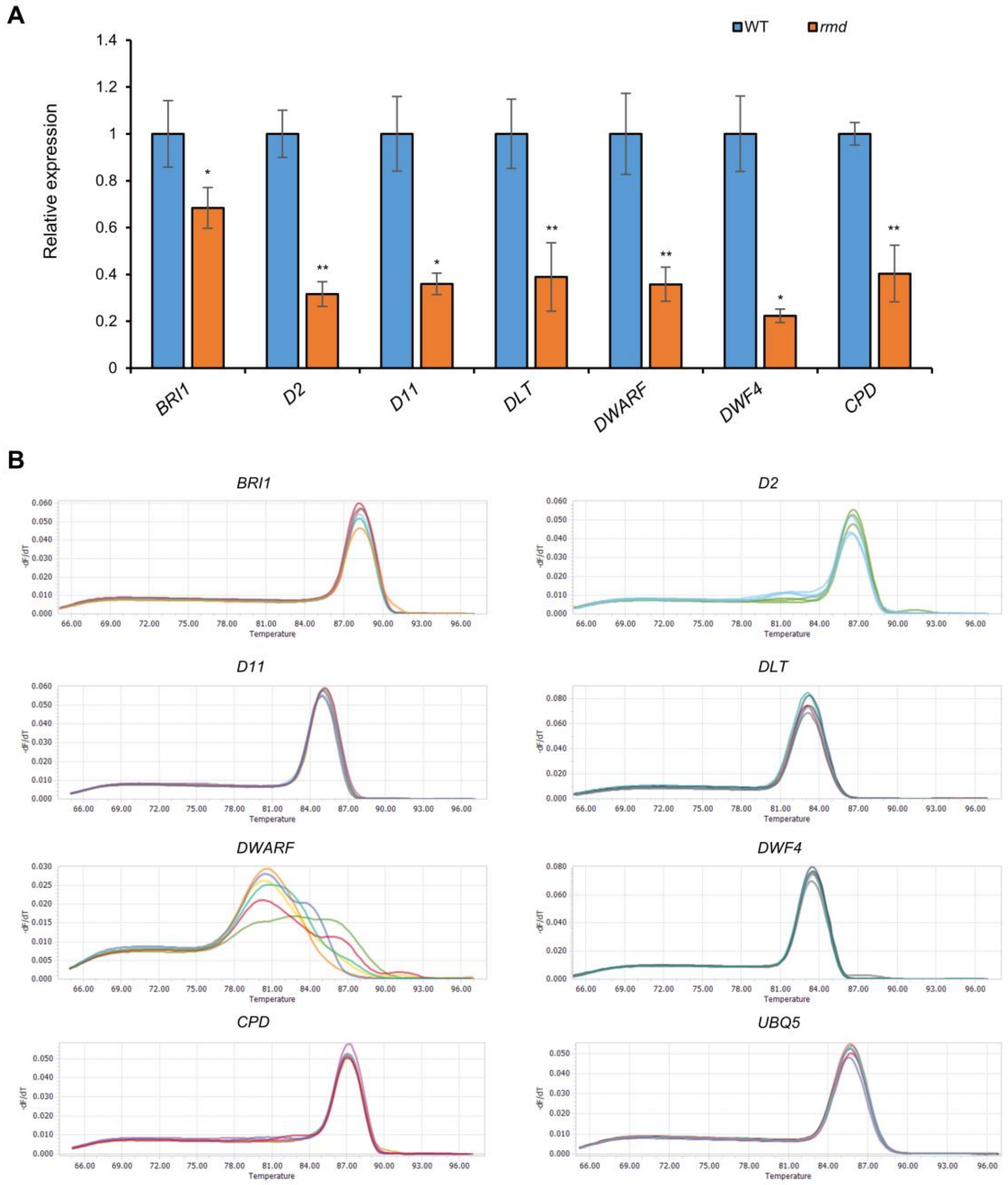

Figure S5. Expression of BR feedback-regulated genes in 7 d WT and *rmd* roots

(A) Relative expression. Data are means ± s.d. (n = 3 replicates); expression relative to *UBQ5*. For each gene, the expression level in WT was set as "1." Asterisks indicate significant differences relative to WT (Student's *t*-test; *$p < 0.05$, **$p < 0.01$). **(B)** Melting curve analysis of qRT-PCR reactions for check of specificity of the primers.

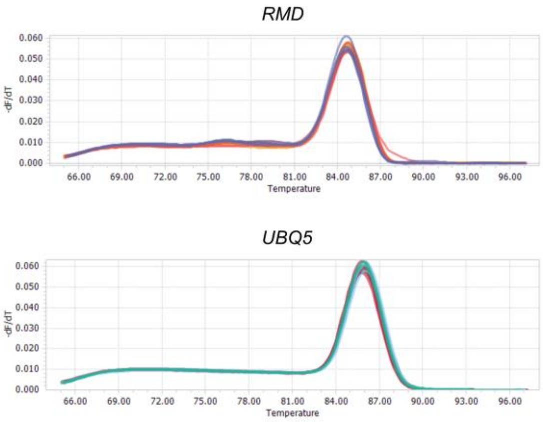

Figure S6. Melting curve analysis of qRT-PCR reactions for check of specificity of the primers used for *RMD* expression analysis under BL treatment in Figure 5C.

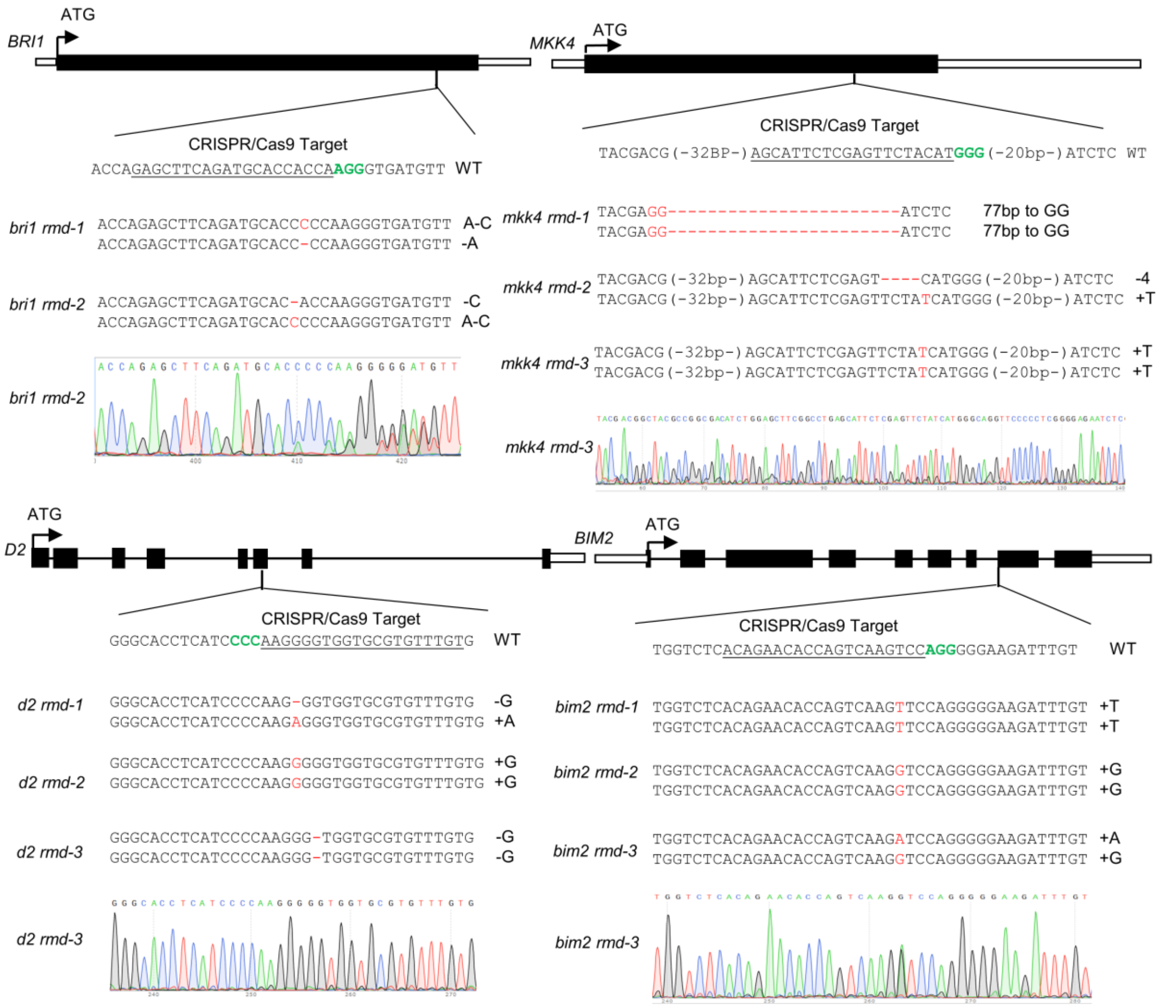

Figure S7. CRISPR/Cas9–induced mutation of *BRI1*, *D2*, *BIM2, MKK4* in the *rmd* background

Black boxes show exons; black lines introns; and open boxes untranslated regions. 20 bp gRNAs are underlined and PAMs highlighted in green. Mutations in resultant lines are highlighted in red.

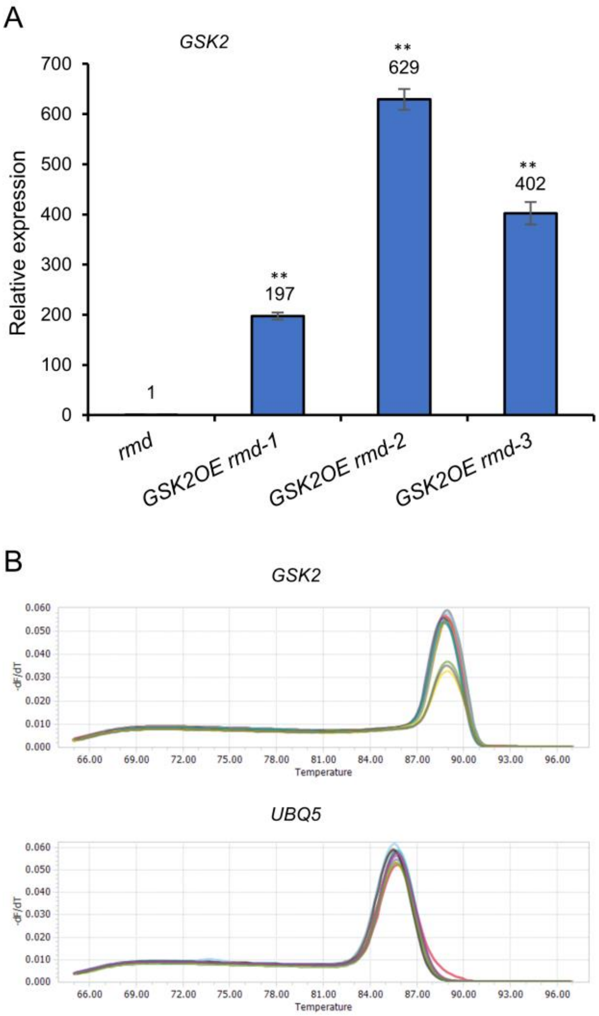

Figure S8. The relative expression of *GSK2* in representative *GSK2* overexpression lines in *rmd* background

(A) Relative expression. Data are means ± s.d. (n = 3 replicates); expression relative to *UBQ5*. The expression level in *rmd* was set as "1." Asterisks indicate significant differences relative to *rmd* (Student's *t*-test; **$p < 0.01$). **(B)** Melting curve analysis of qRT-PCR reactions for check of specificity of the primers.

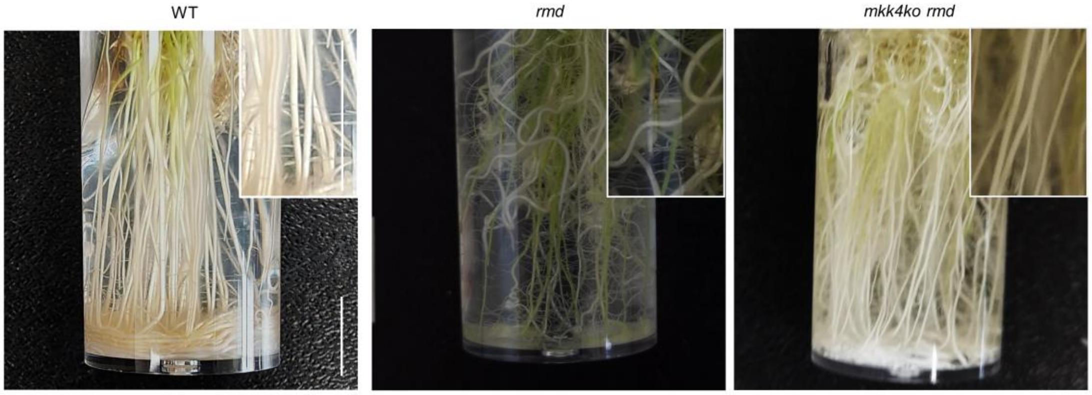

Figure S9. Root phenotypes of WT, *rmd*, and the transgenic T_0 line of *mkk4 rmd* lines

Scale bar, 1 cm.

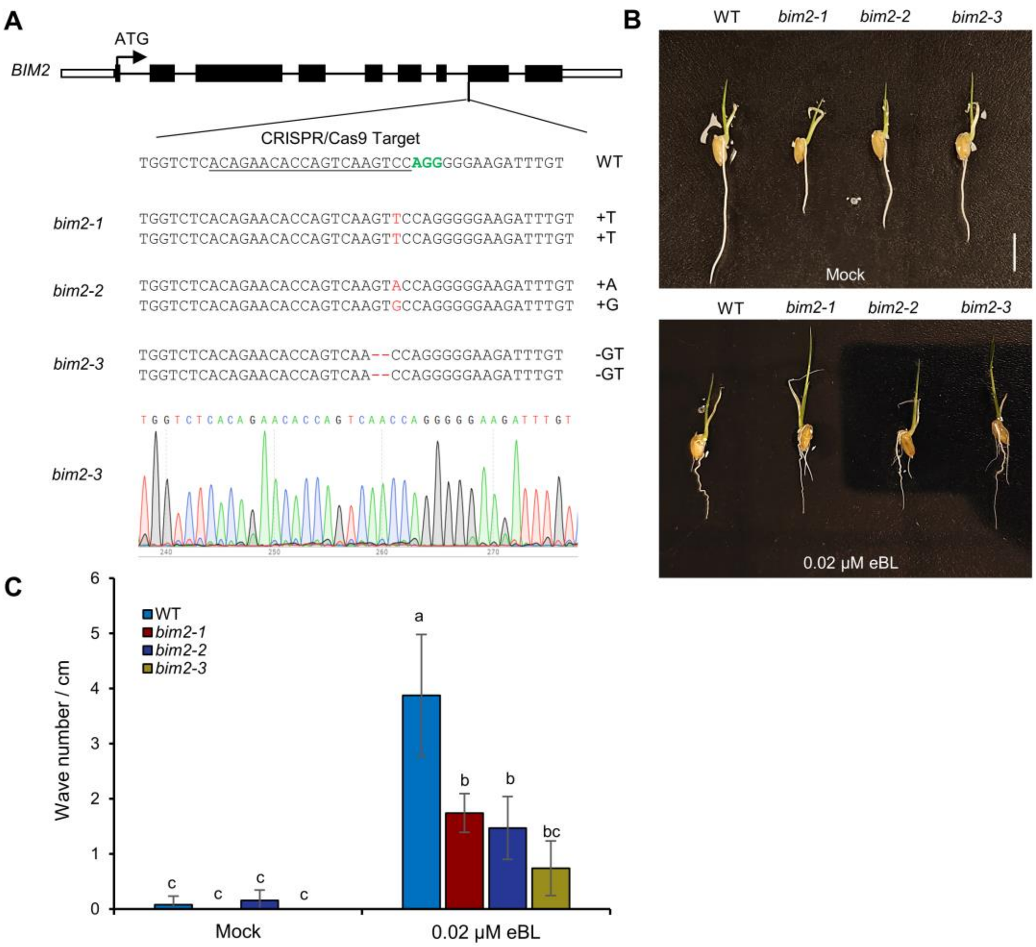

Figure S10. BIM2 positively regulates BR signaling

(A) The genotype of *bim2* lines. Black boxes show exons; black lines introns; and open boxes untranslated regions. 20 bp gRNAs are underlined and PAMs highlighted in green. Mutations in resultant lines are highlighted in red. **(B)** Root of WT and T_1 *bim2* lines after 3 d treatment with and without 0.02 μM eBL. Scale bar, 1 cm. **(C)** Statistic data of wave number per unit root length of WT and T_1 lines of *bim2* knockout lines. Data are means ± s.d. (*n* = 4 biological replicates). Different letters represent significant differences determined by ordinary one-way ANOVA with Tukey's multiple comparisons test, $p < 0.05$).

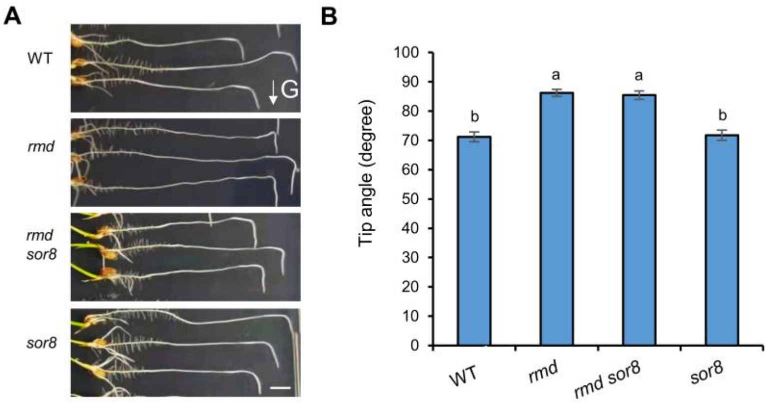

Figure S11. Gravity response of WT, *rmd*, *rmd sor8*, and *sor8* lines

(A) A representative primary root from each line after a 90° gravistimulation. Scale bar, 0.5 cm**.** **(B)** Quantification of tip angle of the root after a 90° gravistimulation**.** Data are means ± s.d. of 3 independent biological replicates, each with $n > 6$ individuals analyzed in each assay. Different letters represent significant differences determined by ordinary one-way ANOVA with Tukey's multiple comparisons test, $p < 0.05$).

Table S1. Primers used in this study

**Primer name**	**Sequence (5'-3')**	**Purpose**
pMAPK6:MAPK6-GFP-F	GAGCTCGGTACCCGGGGATCC<br>CATAAAGCAGGTCCTTTCTAAGGCG	1301-MAPK6-eGFP construct
pMAPK6:MAPK6-GFP-R	GCCCTTGCTCACCATACTAGT<br>CGAGCCACCAGAGCCACC CTGGTAA<br>TCAGGGTTGAACGCAAGG	
GSK2OE-F	AGGTCGACTCTAGAGGATC<br>ATGGACCAGCCGGCGCCG	GSK2OE construct

GSK2OE-R	GCTCTCTAGAACTAGT TTAGCTCCCAGTATTGAAGAAGTTG	
BRI1-T-SENSE	GGCA **A**GAGCTTCAGATGCACCACCA	For CRISPR/Cas9 Knocking out construction, sense and anti-sense oligonucleotides of gRNAs with 4 nt overhangs.The artificially added first nucleotide of gRNAs were shown in bold
BRI1-T-ANTI	AAAC TGGTGGTGCATCTGAAGCTC**T**	
D2-T-SENSE	GGCA ACAAACACGCACCACCCCTT	
D2-T-ANTI	AAAC AAGGGGTGGTGCGTGTTTGT	
MKK4-T-SENSE	GGCA AGCATTCTCGAGTTCTACAT	
MKK4-T-ANTI	AAAC ATGTAGAACTCGAGAATGCT	
BIM2-T-SENSE	GGCA ACAGAACACCAGTCAAGTCC	
BIM2-T-ANTI	AAAC GGACTTGACTGGTGTTCTGT	
RMD-T-SENSE	GGCA **A**CCCAAGGGACTTCCGAGCTG	
RMD-T-ANTI	AAAC CAGCTCGGAAGTCCCTTGGG**T**	
pGBKT7-MAPK6-F	AGAGGAGGACCTGCATATG GACGCCGGGGCGCAG	For Y2H assays plasmid construction, pGBKT7-MAPK6-F and R were used for both BK-MAPK6 and BK-MAPK^{sor8} constructs
pGBKT7-MAPK6-R	GCAGGTCGACGGATCC CTACTGGTAATCAGGGTTGAACGCA	
pGADT7-BIM2-F	ACCAGATTACGCTCATATG CAGCTTTTCCAAGGAGAGGAGC	
pGADT7-BIM2-R	AGCTCGAGCTCGATGGATCC CTAGCTTTTATTGCACCGCCTC	
pGADT7-MKK4-F	ACCAGATTACGCTCATATG CGACCGGGCGGGCCGCCGAG	
pGADT7-MKK4-R	AGCTCGAGCTCGATGGATCC TCATGACGGAGGCGGTGCG	
MAPK6-nYFP-F	GGACGCCGGCGGATCC ATGGACGCCGGGGCGCAGC	For BiFC assays plasmid construction
MAPK6-nYFP-R	GCAGGTCGACTCTAGA CTACTGGTAATCAGGGTTGAACGCAA	
BIM2-cYFP-F	AGGTACCCGGGGATCC ATGCAGCTTTTCCAAGGAGAGG	
BIM2-cYFP-R	CGCCGTCGACTCTAGA GCTTTTATTGCACCGCCTCTTGG	
MKK4-cYFP-F	AGGTACCCGGGGATCC ATGCGACCGGGCGGGCCGCCGAG	
MKK4-cYFP-R	CGCCGTCGACTCTAGA TGACGGAGGCGGTGCGA	
UBQ5-RT-F	ACCACTTCGACCGCCACTACT	Primers for qRT-PCR
UBQ5-RT-R	ACGCCTAAGCCTGCTGGTT	
RMD-RT-F	TAATAGGTAGAGGGCGTGGG	
RMD-RT-R	CTGACAAATCAAACTCCGAAAC	
BRI1-RT-F	CCTTACGTTTATCTTCGCAACG	
BRI1-RT-R	CCGTGCTCCCCACATACATT	

D2-RT-F	CTGATCGGGCTGGAGGCA	
D2-RT-R	GCCTGGAGTGATCTGTAGAGCC	
D11-RT-F	TGCGAGGAGGCAAGAAAGTT	
D11-RT-R	CCTGGGATGTAGAGAGGGAAAG	
DLT-RT-F	GAGCGCCACGAGAGCTTC	
DLT-RT-R	TACTTGTCCGGCCCGAAC	
DWARF-RT-F	GGTGGAACGAGGTGCGGTA	
DWARF-RT-R	CCTGAACACGCTCCCGTAC	
DWF4-RT-F	AGCAGCACATCGCACGGTA	
DWF4-RT-R	CCTCGTTCTGCAGGATGTACC	
CPD-RT-F	CCAGGGTTTGCAGTGAGGC	
CPD-RT-R	TGGGATCATTCCGGTCAGTT	
GSK2-RT-F	GTACGCGGAGGGGAATGAC	
GSK2-RT-R	AGCCTGAAAGACGATACCGAA	
HYG-F	GGCGAAGAATCTCGTGCTTTCA	For transgene detection of OE lines
HYG-R	CAGGACATTGTTGGAGCCGAAA	
Cas9-F	AGATCACAAAGCACGTGGCAC	For transgene detection of knocking out lines
Cas9-R	GCAAAATCCCGGCCCTTA	
gMAPK6-F	GATGCTGACATTTGATCCTAGACAGA	For transgene detection of *pMAPK6:MAPK6-GFP*complementing lines
GFP-R	GGACACGCTGAACTTGTGGC	
sor8-F	GATGCTGACATTTGATCCTAGACAGA	For *rmd* and *sor8* background verification
sor8-R	TCAGGTGAGGCAATTATTTACAACTA	
rmd-F	TCAGTTGTTTCTTTACACTGGGGC	
rmd-R	AAATCATGAACTCACAGTTTCCACC	
BRI1-T-F	AATACATGTGAGCGGGCAGG	For genotyping of knocking out lines in rmd background, T-Ss were used for sequencing
BRI1-T-R	GACCCCATAGCCTCCCTCAT	
BRI1-T-S	GCAGGGTGACCGGGAGTTC	
D2-T-F	TCACATTCTAATGCTACCAGGAGG	
D2-T-R	CACTAATGCAACTTTGAGGGAATC	
D2-T-S	AAGAGGCGAAAAACCGACA	
MKK4-T-F	TCCAACCTCCTCATCGACTCC	
MKK4-T-R	TAATTCCAGGAGCAACAGCGA	
MKK4-T-S	TCAACCAGACCATGGACCCC	
BIM2-T-F	GTCCAAAAGTATGAGGAAGCCG	
BIM2-T-R	CCTGGTTCAGACTCTTGGCG	
BIM2-T-S	TGAGGAAGCCGATCCAGAA	
RMD-T-F	AATAGTTGCAATGCAGGCGGA	
RMD-T-R	CTCATTCTCACACGCCTGCTG	
RMD-T-S	AGTTGGTGCTTTTTGCCGTGT	

Supporting movies legends

Movie S1. Root circumnutation of wild type. Video depicts 3 hours of growth.

Movie S2. Root circumnutation of *rmd*. Video depicts 3 hours of growth.

Movie S3. Root circumnutation of *rmd sor8*. Video depicts 3 hours of growth.

Movie S4. Root circumnutation of *sor8*. Video depicts 3 hours of growth.
